# IL6 trans-signaling associates with ischemic stroke but not with atrial fibrillation

**DOI:** 10.1186/s12883-021-02321-6

**Published:** 2021-08-09

**Authors:** Louise Ziegler, Håkan Wallén, Sara Aspberg, Ulf de Faire, Bruna Gigante

**Affiliations:** 1 Department of Clinical Sciences Karolinska Institutet, Division of Internal Medicine, Danderyd Hospital, S-182 88 Stockholm, Sweden; 2 Department of Clinical Sciences Karolinska Institutet, Division of Cardiovascular Medicine, Danderyd Hospital, Stockholm, Sweden; 3 Unit of Cardiovascular and Nutritional Epidemiology Karolinska Institutet, Stockholm, Sweden; 4 Cardiovascular Medicine Unit, Department of Medicine Karolinska Institutet, Stockholm, Sweden

**Keywords:** Epidemiology, Ischemic stroke, Atrial fibrillation, Inflammation

## Abstract

**Background:**

Pro-inflammatory processes underlie ischemic stroke, albeit it is largely unknown if they selectively associate with the risk of atherothrombotic or cardioembolic ischemic stroke. Here we analyze whether pro-inflammatory interleukin (IL) 6 trans-signaling, is associated with the risk of ischemic stroke and underlying atrial fibrillation (AF).

**Methods:**

During a 20-year follow-up, 203 incident ischemic strokes were recorded from national registers in the cohort of 60-year-old men and women from Stockholm (n = 4232). The risk of ischemic stroke associated with circulating IL6 trans-signaling, assessed by a ratio between the pro-inflammatory binary IL6:sIL6R complex and the inactive ternary IL6:sIL6R:sgp130 complex (B/T ratio), was estimated by Cox regression and expressed as hazard ratio (HR) with a 95% confidence interval (CI) in the presence or absence of AF. Risk estimates were adjusted for cardiovascular risk factors and anticoagulant treatment. In a secondary analysis, the association of IL6 trans-signaling with the risk of incident AF (n = 279) was analyzed.

**Results:**

B/T ratio > median was associated with increased risk of ischemic stroke in study participants without AF (adjusted HR 1.49; 95% CI 1.08–2.06), while an association could not be demonstrated in the presence of AF. Moreover, the B/T ratio was not associated with the risk of AF (HR 0.96; 95% CI 0.75–1.24).

**Conclusions:**

Pro-inflammatory IL6 trans-signaling, estimated by the B/T ratio, is associated with ischemic stroke in individuals without AF. These findings suggest that the B/T ratio could be used to assess the risk of non-AF associated ischemic stroke.

**Supplementary Information:**

The online version contains supplementary material available at 10.1186/s12883-021-02321-6.

## Background

Inflammation driven by interleukin (IL) 6 represents one of the mechanisms underlying different forms of ischemic stroke [1–3]. IL6 signals through two pathways mediating opposing effects. In trans-signaling, IL6 mediates a potent pro-inflammatory and pro-atherogenic effect while classical IL6 signaling entails effects essential to the immune system and tissue homeostasis [4]. In classical signaling, IL6 binds the membrane bound IL6 receptor (IL6R) and the signal transducing receptor, glycoprotein 130 (gp130). In IL6 trans-signaling on the other hand, IL6 binds a soluble IL6R isoform (sIL6R) forming the circulating IL6:sIL6R (binary) complex able to bind and activate gp130. The active binary IL6:sIL6R complex is inhibited by the soluble gp130 (sgp130) through the rapid formation of an IL6:sIL6R:sgp130 (ternary) complex. The ternary complex impedes IL6 trans-signaling by preventing binding to gp130 [5].

IL6 trans-signaling contributes to sustained inflammation in chronic conditions such as atherosclerosis [4, 6] and experimental research indicate that trans-signaling could be detrimental in the ischemic brain [7]. We recently demonstrated that IL6 trans-signaling is associated with the risk of ischemic stroke and the binary/ternary complex (B/T) ratio, improved risk classification measures in individuals otherwise classified as at low-intermediate risk for cardiovascular events [8]. We have also shown that all IL6 trans-signaling components (IL6, sIL6R and sgp130) are expressed in high-grade carotid artery plaques indicating a role for IL6 trans-signaling in large vessel cerebrovascular disease [9].

Biomarkers of IL6 trans-signaling are emerging in cardiovascular risk prediction [6, 10–12]. However, there are no studies addressing their role as predictors of cardioembolic vs. atherothrombotic ischemic stroke. As the preventive treatment modalities in ischemic cerebrovascular disease differ, novel biomarkers able to improve prediction of cardioembolic and atherothrombotic stroke would be of great clinical relevance. Atrial fibrillation (AF), the most common supraventricular arrhythmia is associated with an increased risk of cardiac embolization and ischemic stroke and the inflammatory state described in AF includes higher plasma levels of IL6 associated with an increased risk of secondary thromboembolism and mortality [13–15]. IL6 trans-signaling specifically has however not been studied in relation to AF or cardioembolic stroke risk.

## Aims

We aimed at investigating the role of IL6 trans-signaling in ischemic stroke in relation to AF. The primary aim was to analyze a potential association between IL6 trans-signaling, mirrored by the B/T ratio, and the risk of ischemic stroke in a prospective cohort of middle-aged subjects free of prevalent cardiovascular disease (CVD) with and without AF. The secondary aim was to analyze possible associations between IL6 trans-signaling, and the risk of incident AF.

## Methods

### Study population

In the cohort study of 60-year-old men and women from Stockholm, every third man and woman living in the Stockholm County and turning 60 years in 1997–1998 was invited to participate and, with a 78% positive response rate, 4232 participants were included [6]. At the baseline visit, participants were given a self-administered questionnaire on lifestyle, medical family history, past and chronic diagnoses and current medication. Body weight, height and blood pressure was measured, a 12-lead resting electrocardiogram (ECG) recorded and fasting blood samples drawn in the morning and immediately frozen to -80 degrees Celsius for future analyses. Subjects with signs of infection were rescheduled to a later date to avoid the inflammatory markers from being affected.

Subjects were excluded from the present analysis if they had not filled out the questionnaire (n = 122), lacked serum samples (n = 96), had prevalent coronary or cerebrovascular disease at baseline (n = 369) or incident coronary events during follow-up (n = 433). In addition, participants inaccurately categorized as incident ischemic stroke with the International Classification of Diseases 10th revision (ICD-10) diagnosis codes I649 or I652 (n = 19) were restricted from final analyses leaving 3193 individuals in the primary analysis. For a detailed overview of the exclusions please see Additional Fig. 1.

**Fig. 1:**
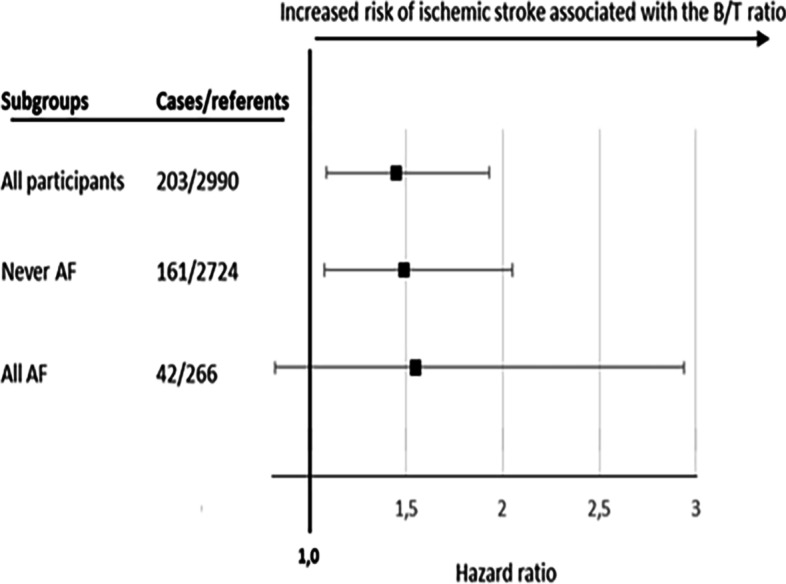
Risk of future ischemic stroke associated with the B/T ratio > median in subjects with and without a diagnosis of AF analyzed by Cox regression and expressed as hazard ratio with 95% confidence interval. Adjustments were made for sex, smoking, hypertension, hyperlipidemia, diabetes mellitus, BMI, and antithrombotic treatment

### Outcome ascertainment

Study participants were followed up until December 31^st^, 2017 via their personal identification numbers through linkage to the Swedish national registers; The Swedish National Inpatient Register with a 100% capture of all hospitalized patients in Sweden and The National Cause of Death Register recording all deaths and cause of death diagnoses in Sweden. Primary diagnoses of non-fatal and fatal ischemic stroke (I63) were registered. In secondary analyses, main and secondary diagnoses of incident AF (I48) were registered to assess incident AF. The ICD-10 code I489 includes atrial flutter, i.e. cases of atrial flutter were included. To analyze the risk of incident AF, subjects with prevalent AF were excluded (n = 29) as were incident ischemic stroke cases (n = 198) due to the well-known high proportion of undiagnosed AF in this group (Additional Fig. 1).

### Biochemical measurements and derivation of the binary and ternary complex molar concentrations

Baseline serum levels of IL6, sIL6R and sgp130 were analyzed as described in the Additional files and in a prior publication [6]. The binary (IL6:sIL6R) and ternary (IL6:sIL6R:sgp130) complexes were estimated from their molar concentrations with formulas previously presented [6, 16, 17]. The ratio between the binary and ternary complex, B/T ratio was calculated for each individual.

### Statistical analysis

Continuous variables are presented as median and interquartile range (IQR) while binary variables are presented as numbers and percentages.

The risk of ischemic stroke associated with IL6 trans-signaling estimated by the B/T ratio was analyzed by Cox proportional hazards model. The risk estimates are given as hazard ratios (HR) with 95% confidence intervals (CI). In the primary analysis, the risk of ischemic stroke was analyzed in subgroups defined by the presence or absence of AF (prevalent or incident). The results from the primary analysis are presented in a crude model and after adjustment for the common cardiovascular risk factors identified at baseline: sex, body mass index (BMI) expressed as kg/m^2^, hypertension (self-reported, or blood pressure > 140/90 mm Hg recorded at the baseline visit), diabetes mellitus (self-reported, or fasting glucose > 7.0 mmol/L in the baseline test), hypercholesterolemia (self-reported or fasting total cholesterol > 5.0 mmol/L), smoking, and chronic treatment at baseline with anticoagulant drugs with codes from the Anatomic Therapeutic Chemical classification system (ATC): B01AA (vitamin K antagonists) or B01AB (heparin group).

In secondary analyses, the risk of incident AF associated with IL6 trans-signaling was analyzed, including each of the IL6 trans-signaling components IL6, sIL6R, sgp130, and the B/T ratio categorized in quartiles or dichotomized at the median. The secondary analysis is presented in a crude model and in a model adjusted for sex, hypertension, BMI, and left ventricular hypertrophy defined as the presence of either one of two established ECG criteria, the Minnesota Code or Cornell voltage-duration product.

To account for the effect of age on the risk of AF, the cumulative AF incidence was also presented graphically using Kaplan Meier curves and stratified by the B/T ratio dichotomized at the median and in additional analyses stratified by quartiles of IL6, sIL6R and sgp130 and for IL6 also dichotomized at the 75^th^ percentile. Log-rank test was used to test for equality in the survivor functions. To estimate the difference in time to AF diagnosis, quantile regression for censored data was implemented using Laplace regression analysis, expressed in years with 95% CI and adjusted for the above-mentioned confounders.

All statistical analyses were performed on Stata statistical software, Release 14. College Station, TX: StataCorp LP.

## Results

In Table 1, the clinical characteristics of the study populations are presented stratified by the occurrence of an ischemic stroke during follow-up or not. As expected, those that suffered an ischemic stroke carried a greater cardiovascular risk burden than those who did not. Moreover, stroke cases had a significantly higher B/T ratio at baseline compared to non-cases (p = 0.0003).

**Table 1:** Baseline characteristics of the study population stratified by ischemic stroke

**Baseline clinical characteristics**	**Ischemic stroke** (n = 203)	**No ischemic stroke** (n = 2990)
Male (%)	116 (57.1)	1309 (43.8)
Hypertension (%)	45 (22.2)	440 (14.7)
Hyperlipidemia (%)	6 (3.0)	103 (3.4)
Diabetes mellitus (%)	10 (4.9)	76 (2.5)
Atrial fibrillation (%)	5 (2.5)	23 (0.8)
Smoking (%)	56 (27.9)	589 (20.0)
Anticoagulant treatment (%)	1 (0.5)	11 (0.4)
BMI	26.5 (24.4–28.6)	26.0 (23.7–28.8)
Systolic blood pressure	143.5 (130.5–156.5)	134.5 (120.5–150)
Diastolic blood pressure	88 (81–94.5)	82.5 (76–90)
Glucose	5.2 (4.8–5.7)	5.2 (4.8–5.6)
LDL	4.1 (3.4–4.7)	3.8 (3.2–4.5)
B/T ratio	1.60 (1.56–1.63)	1.58 (1.55–1.61)

Continuous variables are presented as median (IQR). BMI is expressed as kg/m^2^, blood pressure as mm Hg, glucose and LDL as mmol/L. Missing data: Smoking (n = 40), LDL (n = 37), and blood pressure (n = 3)

During an approximately 20-year follow-up, there were 203 fatal and non-fatal cases of ischemic stroke. Prevalent AF was registered in 29 study participants at baseline and incident AF in 279 participants during follow-up. Two of the participants with AF at baseline were on warfarin prophylaxis and five suffered an ischemic stroke during follow-up. Of the incident AF cases, 161 were diagnosed with AF as a main diagnosis, 116 as a secondary diagnosis and two had an AF diagnosis not categorized as main or secondary. Study participants with AF (prevalent or incident) suffered more ischemic strokes during follow-up than those without AF while levels of the B/T ratio did not differ (Table 2).

**Table 2:** Number of ischemic strokes and B/T ratio level in subjects with and without atrial fibrillation

	**Never AF**	**AF**	**P**
Total number	2885	308	-
Stroke (%)	161 (5.6)	42 (13.6)	< 0.001
B/T ratio	1.58 (1.55–1.61)	1.58 (1.55–1.61)	0.94

Number of participants with ischemic stroke during follow-up (%) and levels of the B/T ratio presented as median (IQR) in subjects with AF (prevalent or incident) compared to those without (Never AF)

### Risk of future ischemic stroke associated with the B/T ratio in subjects with and without atrial fibrillation

Figure 1 presents graphically the risk of future ischemic stroke associated with the B/T ratio > median (1.58) in subjects without AF (adjusted HR 1.49; 95% CI 1.08–2.06) and in those with prevalent or incident AF (adjusted HR 1.54; 95% CI 0.81–2.91). Crude and adjusted risk estimates are presented in Additional Table 1.

### Risk of incident atrial fibrillation and time to atrial fibrillation associated with the B/T ratio and IL6 trans-signaling components

**Fig. 2:**
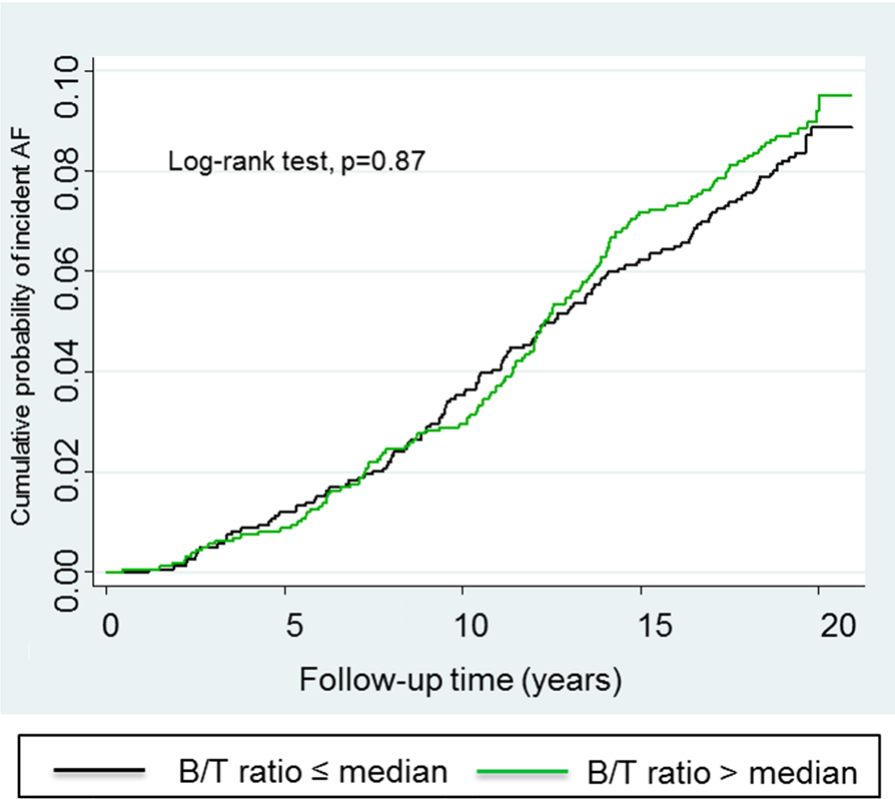
Cumulative incidence of AF in subjects without prevalent AF at baseline stratified by the B/T ratio dichotomized at the median

There was no increased risk of incident AF associated with the B/T ratio categorized into quartiles or dichotomized at the median (Table 3). There was no difference in cumulative incidence of AF when stratifying by the B/T ratio cut at the median, p = 0.87 (Fig. 2).

**Table 3:** Risk of incident atrial fibrillation associated with the B/T ratio

B/T ratio	Crude	P	Adjusted	P
≤ 25^th^ perc	1.00 (ref)	-	1.00 (ref)	-
25-50^th^ perc	0.90 (0.63–1.29)	0.56	0.84 (0.59–1.21)	0.35
50-75^th^ perc	1.00 (0.71–1.43)	0.98	0.91 (0.64–1.29)	0.60
> 75^th^ perc	0.98 (0.69–1.40)	0.92	0.86 (0.60–1.23)	0.40
≤ median	1.00 (ref)	-	1.00 (ref)	-
> median	1.05 (0.81–1.35)	0.73	0.96 (0.75–1.24)	0.77

Risk of incident AF associated with the B/T ratio, categorized into percentiles (perc) and dichotomized at the median, analyzed by Cox regression and expressed as HR (95% CI). Multivariate analysis adjusted for sex, hypertension, BMI, and left ventricular hypertrophy. Participants with prevalent AF at baseline were excluded from this analysis. Missing data on left ventricular hypertrophy (n = 6)

Investigating the association with IL6 signaling further, there was a borderline significant association between the risk of AF and IL6 > 75^th^ percentile (HR 1.23; CI 0.93–1.63), as seen in Additional Table 2. In addition, a pattern of earlier AF diagnosis associated with high IL6 levels was seen in Kaplan Meier curves (Additional Fig. 2). At the end of follow-up, when adjusting for the above-mentioned confounders (sex, BMI, smoking, hypertension, diabetes, hypercholesterolemia and chronic treatment at baseline with anticoagulants), participants with IL6 levels > 75^th^ percentile were diagnosed with AF 4.3 years earlier (95% CI 1.1–7.5 years, p = 0.008) compared to those with lower levels. No association with increased risk of AF or earlier AF diagnosis was observed for the soluble trans-signaling receptors, sIL6R or sgp130 (Additional Table 3, Additional Fig. 3).

## Discussion

Here we demonstrate that IL6 trans-signalling, mirrored by the B/T ratio > median, is associated with increased risk of ischemic stroke albeit the association could not be shown in the small group of participants with AF nor with the risk of AF specifically.

Increased IL6 levels in the circulation and in the central nervous system have been demonstrated in the acute phase of ischemic stroke [18, 19]. The role of IL6 is however controversial with evidence of both detrimental and protective properties in ischemic stroke models and no effect on infarct size or neurological function in IL6 knock-out mice [20, 21].

We previously demonstrated that the B/T ratio, a biomarker assessing the pro-inflammatory IL6 trans-signaling pathway, could predict an increased risk of incident ischemic stroke, pre-eminently in individuals with low LDL cholesterol [8]. Moreover, in a small “real-world” cohort of patients with carotid artery stenosis undergoing carotid endarterectomy (n = 78), we analyzed the expression of *IL6* and IL6 receptor genes in carotid artery plaques and found that their expression were upregulated in patients with a recent ischemic cerebrovascular event (≤ 6 months from carotid surgery) [9]. Our former and current data suggest that the IL6 trans-signaling pathway is mainly associated with atherosclerosis related cerebrovascular disease as we could not demonstrate an association with the risk of AF or an increased risk of ischemic stroke in subjects with AF. Of note is that the prevalence and risk factors for AF overlap those for large and small vessel cerebrovascular disease i.e. the underlying pathophysiology is not by default cardioembolic in individuals with AF [22]. A possible implication of our findings is however that the B/T ratio may represent a biomarker to identify individuals at risk for atherothrombotic but not cardioembolic stroke.

Results from Mendelian randomization studies indicate a causal association between the *IL6R*, AF and ischemic stroke and that the increased risk of ischemic stroke associated with the *IL6R* is accounted for by AF [23, 24]. A more recent Mendelian randomization study on the other hand found an association between *IL6R* and the risk of large vessel stroke but not cardioembolic stroke [25]. Nevertheless, these studies do not consider the differential role of IL6 classical and IL6 trans-signaling in the modulation of inflammatory processes, and that the synthesis and release of sIL6R and sgp130 is regulated at a post-transcriptional level. Whether IL6 classical or IL6 trans-signaling is associated with AF or not has not been analyzed in epidemiological studies to this date. Our data suggest that IL6 is possibly associated with an increased risk of AF and that this association is not mediated by IL6 trans-signaling, but potentially driven by the IL6 classical signaling. The observation that there was no difference in the B/T ratio with regards to AF, as well as the fact that neither sIL6R nor sgp130 serum levels were associated with the risk of AF; suggest that the stroke risk associated with the B/T ratio is independent of AF.

### Strengths and limitations

This is the first prospective population-based cohort study exploring the association between the pro-inflammatory IL6 trans-signaling and ischemic stroke in relation to AF. The unique Swedish personal identification number and mandatory reporting of inpatient and hospital-based outpatient diagnoses in national Swedish registers enable a 100% follow-up. With an overall positive predictive value of diagnoses in these registers of 85–95%, diagnoses may be considered reliable.

One major limitation in the study is the lack of diagnoses from primary care in the Swedish inpatient registers. In relation to ischemic stroke this is a marginal problem as stroke is mainly diagnosed in hospitals. AF however is also diagnosed in primary care. To account for this, we have recorded both main and secondary diagnoses of AF, but we cannot entirely compensate for the lack of primary care diagnoses. In addition, as AF in the early stages of the disease is paroxysmal and often asymptomatic, potential delays in diagnosing the condition is impending and thus the first recorded diagnosis is not always equal to the first presentation of the disease. Furthermore, we did not perform long-term ECG recordings in the study, possibly missing paroxysmal AF which it problematic as even subclinical atrial tachyarrhythmias are associated with an increased risk of ischemic stroke [26]. In addition, we did not have enough power to analyze differences in association between pre- and post-stroke AF. Moreover, in the present study we aimed at analyzing the risk of ischemic stroke associated with IL6 trans-signaling in individuals with and without AF. Without access to information on findings from computer tomography/magnetic resonance imaging, carotid ultrasound, long-term ECG recordings, echocardiogram, etc. we were forced to use the composite outcome of ischemic stroke despite its heterogeneous pathophysiological mechanisms and can thus merely speculate on whether the association seen is primarily driven by atherothrombotic rather than cardioembolic origin.

Misclassification would however lead to an underestimation of AF prevalence and incidence and cardioembolic strokes in our cohort and would marginally affect our results: misclassification of ischemic stroke as atherothrombotic could possibly dilute the observed risk since the B/T ratio was associated neither with AF nor with ischemic stroke in AF patients. The non-significant association with stroke risk in the smaller group of participants with prevalent/incident AF could however be due to lack of power albeit the risk estimate was comparable to that in the non-AF group.

In addition, we did not have access to information on prospective changes in medication. With the primary aim to analyze the B/T ratio as a predictive marker of ischemic stroke this is however not mandatory.

Moreover, we only have serum samples from baseline and thus cannot exclude that serum levels at baseline do not mirror the levels during follow-up. In addition, the cut-offs for the B/T ratio and IL6 are data driven which can prevent the results from being generalizable. The aim of the study is however not to find suitable reference values but to analyze associations between IL6 trans-signaling and ischemic stroke. On the other hand, we believe that the data derived from this cohort, being a population-based cohort with participants randomly chosen and with a high positive response rate (78%), are representable in the context of other populations with comparable lifestyle and societal structure.

Finally, the study is observational preventing any mechanistic conclusions from being drawn. The experimental evidence underlying the hypothesis of this study is however in line with the present findings.

## Conclusions

IL6 trans-signaling, assessed by the B/T ratio, is associated with an increased risk of ischemic stroke in patients without known AF. The results suggest the B/T ratio could be a novel biomarker for a more personalized ischemic stroke risk assessment.

## Supplementary Information

**Additional file 1.**

## Data Availability

The datasets used and/or analyzed during the current study are available from the corresponding author on reasonable request.

## Abbreviations

AF: Atrial fibrillation
ATC: Anatomic Therapeutic Chemical classification system
BMI: Body mass index
B/T ratio: Binary/ternary complex ratio
CI: Confidence interval
CVD: Cardiovascular disease
ECG: Electrocardiogram
gp130: Glycoprotein 130
HR: Hazard ratio
ICD-10: International Classification of Diseases 10th revision
IL: Interleukin
IL6R: Interleukin 6 receptor
IQR: Interquartile range
sgp130: Soluble glycoprotein 130
sIL6R: Soluble interleukin 6 receptor
