# Correction: IL6 trans-signaling associates with ischemic stroke but not with atrial fibrillation

**DOI:** 10.1186/s12883-022-02831-x

**Published:** 2022-09-07

**Authors:** Louise Ziegler, Håkan Wallén, Sara Aspberg, Ulf de Faire, Bruna Gigante

**Affiliations:** 1 Department of Clinical Sciences Karolinska Institutet, Division of Internal Medicine, Danderyd Hospital, S-182 88 Stockholm, Sweden; 2 Department of Clinical Sciences Karolinska Institutet, Division of Cardiovascular Medicine, Danderyd Hospital, Stockholm, Sweden; 3 Unit of Cardiovascular and Nutritional Epidemiology Karolinska Institutet, Stockholm, Sweden; 4 Cardiovascular Medicine Unit, Department of Medicine Karolinska Institutet, Stockholm, Sweden

**Correction: BMC Neurol 21, 306 (2021)**

**https://doi.org/10.1186/s12883-021-02321-6**

Following publication of the original article [1], the authors reported an error in the number of incident atrial fibrillation cases resulting in errors in Tables 2 and 3, Figs. 1 and 2, and the Supplemental Material. The correct tables, figures and Supplemental Material are given below.

**Table 2:** Number of ischemic strokes and B/T ratio level in subjects with and without atrial fibrillation

	Never AF	AF	P
Total number	2748	445	-
Stroke	140 (5%)	63 (14%)	<0.0001
B/T ratio	1.58 (1.54-1.61)	1.58 (1.54-1.61)	0.94

Number of participants with ischemic stroke during follow-up (%) and levels of the B/T ratio presented as median (IQR) in subjects with AF (prevalent or incident) compared to those without (Never AF)

**Table 3:** Risk of incident atrial fibrillation associated with the B/T ratio

B/T ratio	Crude	P	Adjusted	P
≤25^th^ perc	1.00 (ref)	-	1.00 (ref)	
25-50^th^ perc	0.93 (0.71-1.23)	0.65	0.89 (0.67-1.17)	0.41
50-75^th^ perc	1.01 (0.77-1.32)	0.93	0.92 (0.70-1.21)	0.57
>75^th^ perc	1.00 (0.77-1.32)	0.94	0.86 (0.65-1.14)	0.30
≤median	1.00 (ref)	-	1.00 (ref)	
>median	1.04 (0.86-1.26)	0.67	0.94 (0.78-1.15)	0.59

Risk of incident AF associated with the B/T ratio, categorized into percentiles (perc) and dichotomized at the median, analyzed by Cox regression and expressed as HR (95% CI). Multivariate analysis adjusted for sex, hypertension, BMI, and left ventricular hypertrophy. Participants with prevalent AF at baseline were excluded from this analysis. Missing data on left ventricular hypertrophy (*n* = 6)

**Fig. 1:**
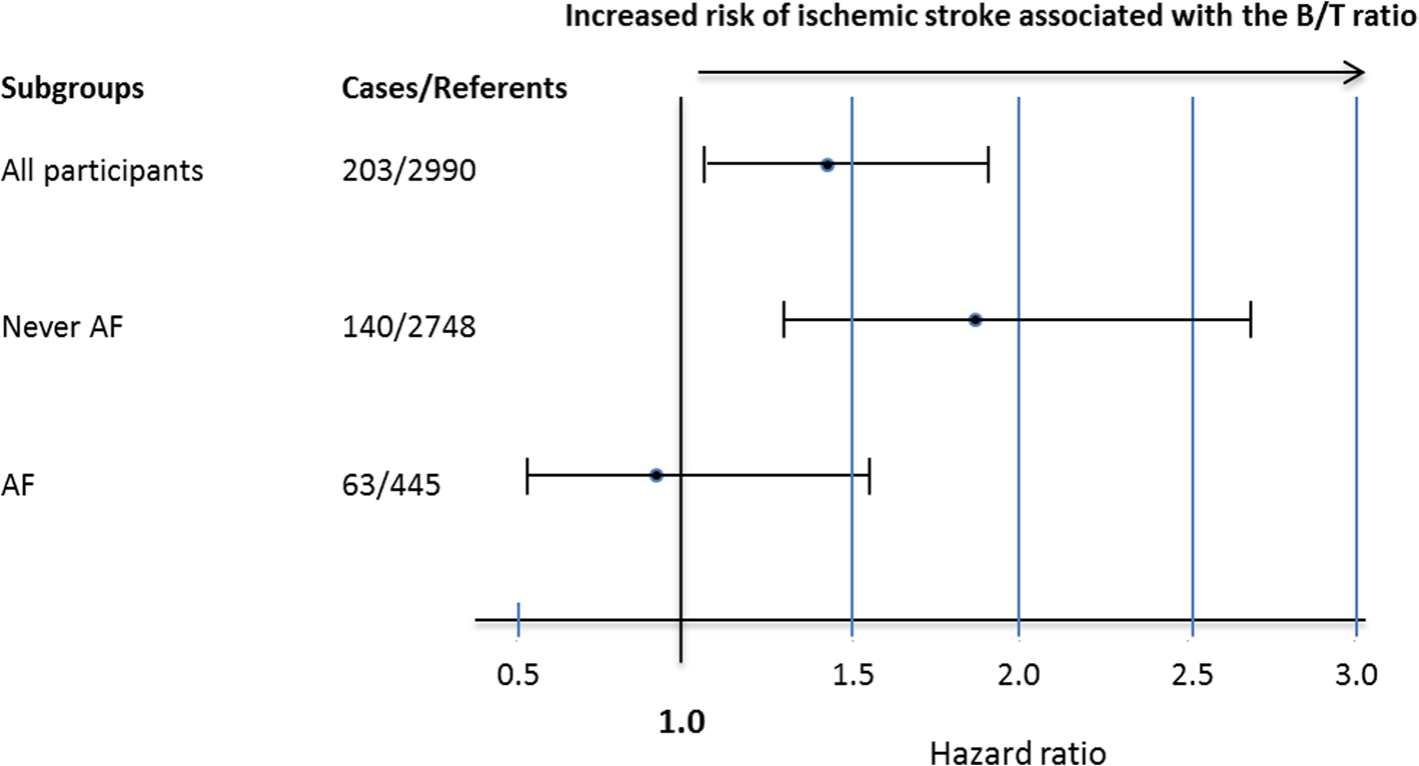
Risk of future ischemic stroke associated with the B/T ratio > median in subjects with and without a diagnosis of AF analyzed by Cox regression and expressed as hazard ratio with 95% confidence interval

**Fig. 2:**
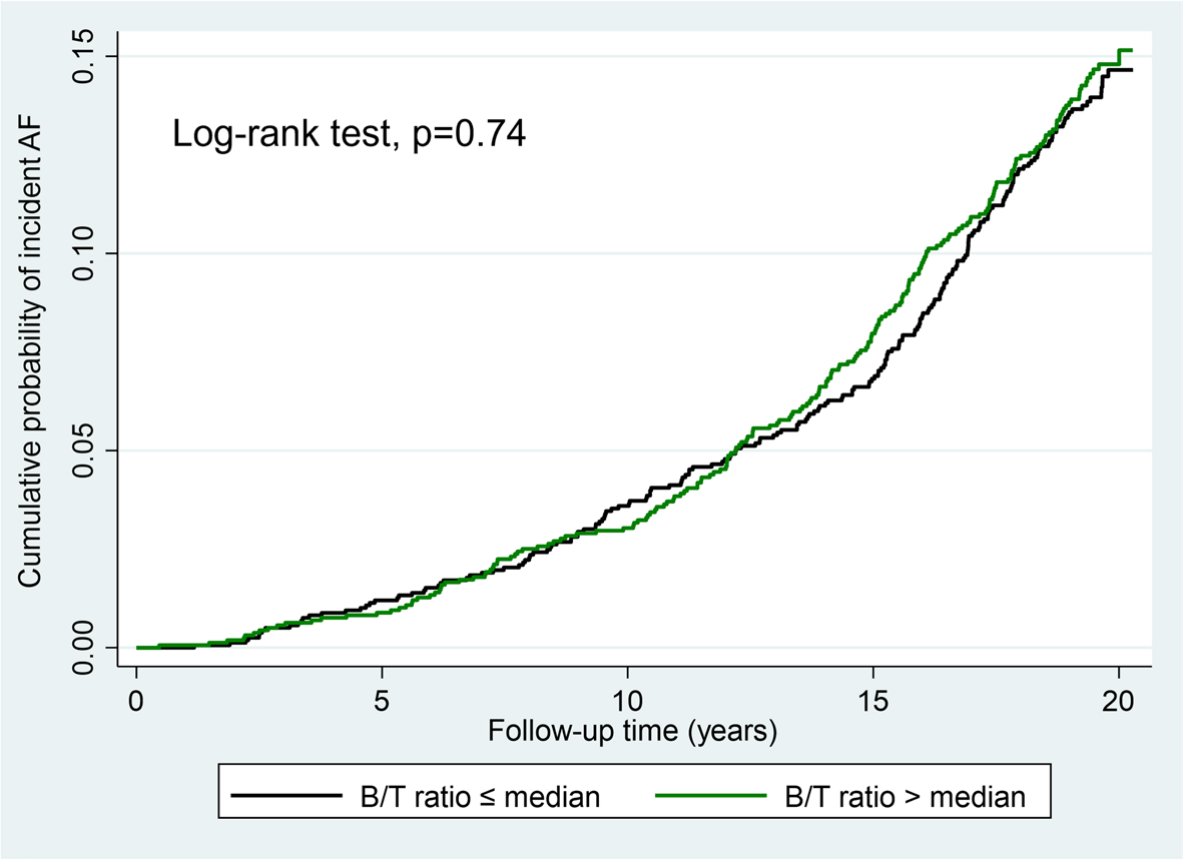
Cumulative incidence of AF in subjects without prevalent AF at baseline stratifed by the B/T ratio dichotomized at the median

The original article [1] has been updated.

## Supplementary Information

**Additional file 1.**
