# Supplementary material for: Correction: IL6 trans-signaling associates with ischemic stroke but not with atrial fibrillation
Source: BMC Neurol. 2022 Sep 7;22:337. doi: 10.1186/s12883-022-02831-x (PMC9450387; doi:10.1186/s12883-022-02831-x)
Supplement: Supplementary file 1 — Additional file 1. [file 12883_2022_2831_MOESM1_ESM.docx]

# Supplementary Material

# IL6 trans-signaling associates with ischemic stroke but not with atrial fibrillation

Louise Ziegler MD, PhD ^1^; Håkan Wallén MD, PhD ^2^; Sara Aspberg MD, PhD ^2^; Ulf de Faire MD, PhD ^3^; Bruna Gigante MD, PhD FESC ^4^.

^1^ Department of Clinical Sciences Karolinska Institutet; Division of Internal Medicine, Danderyd Hospital ^2^ Department of Clinical Sciences Karolinska Institutet; Division of Cardiovascular Medicine, Danderyd Hospital ^3^ Unit of Cardiovascular and Nutritional Epidemiology, IMM, Karolinska Institutet ^4^ Cardiovascular Medicine Unit, Department of Medicine, Karolinska Institutet, Stockholm Sweden.

**Corresponding author contact information:**

Louise Ziegler MD, PhD

Karolinska Institutet, Department of Clinical Sciences

Danderyd Hospital S-182 88 Stockholm, Sweden

Tel: +46 8 123 580 48/550 00, Fax: +46 8 524 800 00

E-mail: [louise.dencker-ziegler@sll.se](mailto:louise.dencker-ziegler@sll.se)

**Content:**

**Additional Material and Methods**

Additional Figure 1

**Additional Results**

Additional Table 1-3

Additional Figure 2-3

***Additional*** ***Material and Methods***

### **Additional** Figure 1. Flow chart of exclusions for the primary and secondary analysis

**
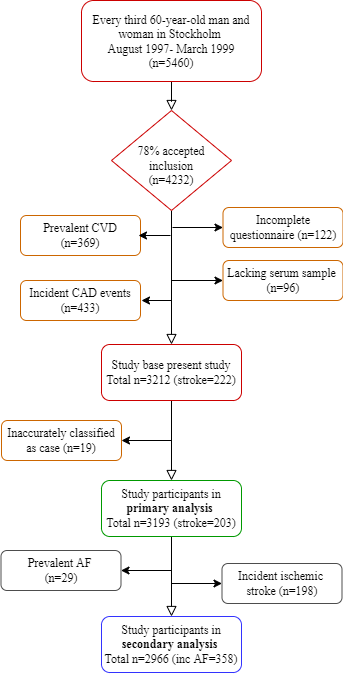
**

**Additional** Figure 1. Participants were excluded if they had not completed the study questionnaire (n=122), had prevalent cardiovascular disease (CVD) at baseline i.e., reported a history of prior ischemic coronary or cerebrovascular event in the questionnaire or if either one of the following ICD-10 diagnosis codes: I21, I25 or I63 were recorded in the National Inpatient Register (n=369). In addition, participants lacking serum sample at the time of analysis (n=96) were excluded as were those with an incident coronary artery disease (CAD) event during follow-up with either of the following ICD-10 diagnosis codes: I21, I25 or I46 (n=433). In addition, 19 participants categorized as cases of incident ischemic stroke with the following ICD-10 codes I652, I64 were restricted from the primary analysis due to ambiguity of their ischemic stroke case status. For the secondary analysis, estimating risk of incident atrial fibrillation (inc AF), study participants with prevalent AF were excluded (n=29) and in addition subjects with incident ischemic stroke during follow-up were excluded (n=203 out which n=198 did not overlap with prevalent AF) due to the overrepresentation of undiagnosed AF in this group of patients. Stroke = ischemic stroke.

### **Biochemical measurements**

Before analyses, serum samples were diluted: IL6 1:2, sIL6R 1:50 and sgp130 1:100. IL6 and sIL6R were then analyzed with the Mesoscale Discovery Systems Cytokine Assay (Gaithersburg, MD, USA) and sgp130 with a development kit assay (#DY228) from R&D Systems ® (R&D systems Minneapolis, MN, USA). Concentrations, reported in pg/mL (IL6) and ng/mL (sIL6R and sgp130), were derived from the experimental standard curve. All experiments were performed according to manufacturer’s protocols. The experimental procedures, including intra- and inter-assay variability, have been described in detail previously 1.

### **Derivation of the molar concentrations of the IL6 complexes**

The mass concentration of each molecule incorporated in the two IL6 complexes was converted into a molar concentration. Using a formula, originally presented by Müller-Newen and Garbers, the molar concentrations of the binary and ternary complex was estimated 1-3.

## *Additional Results*

### **Additional Table 1.** Risk of ischemic stroke associated with the B/T ratio in subjects with and without AF

|  | **Cases/ref** | **Crude** | **P** | **Adjusted** | **P** |
| --- | --- | --- | --- | --- | --- |
| **Never AF** | 140/2748 | 2.12 (1.49-3.02) | <0.0001 | 1.87 (1.31-2.68) | 0.001 |
| **AF** | 63/445 | 0.85 (0.52-1.40) | 0.54 | 0.92 (0.55-1.54) | 0.77 |

**Additional Table 1.** Risk of future ischemic stroke associated with the B/T ratio >median in

subjects with and without AF (Never AF) analyzed by Cox regression and expressed as HR

(95% CI). Case=cases of ischemic stroke and ref=referent group, i.e., without stroke during

follow-up. Individuals with AF were excluded from the analysis in the never AF group.

Multivariate analysis adjusted for sex, smoking, hypertension, hyperlipidemia, diabetes

mellitus, BMI, and anticoagulant treatment.

### **Additional Table 2.** Risk of incident atrial fibrillation associated with IL6

| **IL6** | **Crude** | **P** | **Adjusted** | **P** |
| --- | --- | --- | --- | --- |
| ≤25^th^ perc | 1.00 (ref) | - | 1.00 (ref) | - |
| 25-50^th^ perc | 1.17 (0.89-1.54) | 0.24 | 1.05 (0.79-1.38) | 0.72 |
| 50-75^th^ perc | 1.04 (0.78-1.38) | 0.77 | 0.85 (0.63-1.13) | 0.28 |
| >75^th^ perc | 1.36 (1.04-1.78) | 0.024 | 1.06 (0.80-1.42) | 0.47 |
| ≤75^th^ perc | 1.00 (ref) | - | 1.00 (ref) | - |
| >75^th^ perc | 1.27 (1.02-1.57) | 0.027 | 1.11 (0.89-1.39) | 0.33 |

**Additional Table 2.** Risk of incident atrial fibrillation (AF) associated with IL6, categorized into percentiles (perc) and dichotomized at the 75^th^ percentile, analyzed by Cox regression and expressed as HR (95% CI). Multivariate analysis adjusted for sex, hypertension, BMI, and left ventricular hypertrophy. Participants with prevalent AF at baseline were excluded from the present analysis. Missing data on left ventricular hypertrophy (n=6).

### **Additional Table 3.** Risk of incident atrial fibrillation associated with sIL6R and sgp130

|  | **Crude** | **P** | **Adjusted** | **P** |
| --- | --- | --- | --- | --- |
| **sIL6R** |  |  |  |  |
| 25-50^th^ perc | 0.99 (0.75-1.31) | 0.98 | 0.93 (0.71-1.23) | 0.65 |
| 50-75^th^ perc | 1.09 (0.83-1.42) | 0.53 | 0.96 (0.73-1.27) | 0.82 |
| >75^th^ perc | 1.03 (0.79-1.36) | 0.79 | 0.85 (0.64-1.13) | 0.27 |
| **sgp130** |  |  |  |  |
| 25-50^th^ perc | 1.27 (0.96-1.69) | 0.08 | 1.18 (0.89-1.57) | 0.23 |
| 50-75^th^ perc | 1.41 (1.1-1.85) | 0.01 | 1.20 (0.91-1.60) | 0.18 |
| >75^th^ perc | 1.26 (0.95-1.67) | 0.10 | 1.07 (0.80-1.42) | 0.64 |

**Additional Table 3.** Risk of incident atrial fibrillation (AF) associated with sIL6R and sgp130, categorized into percentiles (perc), analyzed by Cox regression and expressed as HR (95% CI). Multivariate analysis adjusted for sex, hypertension, BMI and left ventricular hypertrophy. Participants with prevalent AF at baseline were excluded from the present analysis. Missing data on left ventricular hypertrophy (n=6).

### **Additional Figure 2.** Cumulative incidence of atrial fibrillation stratified by IL6


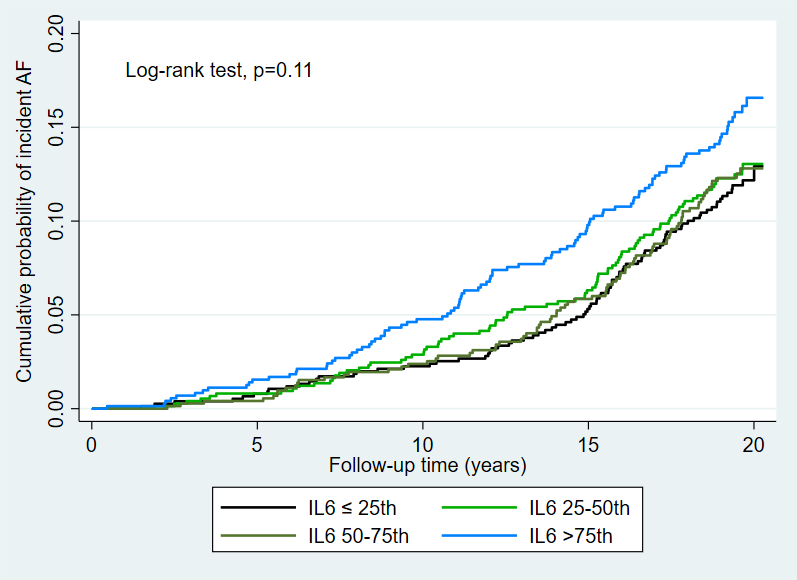

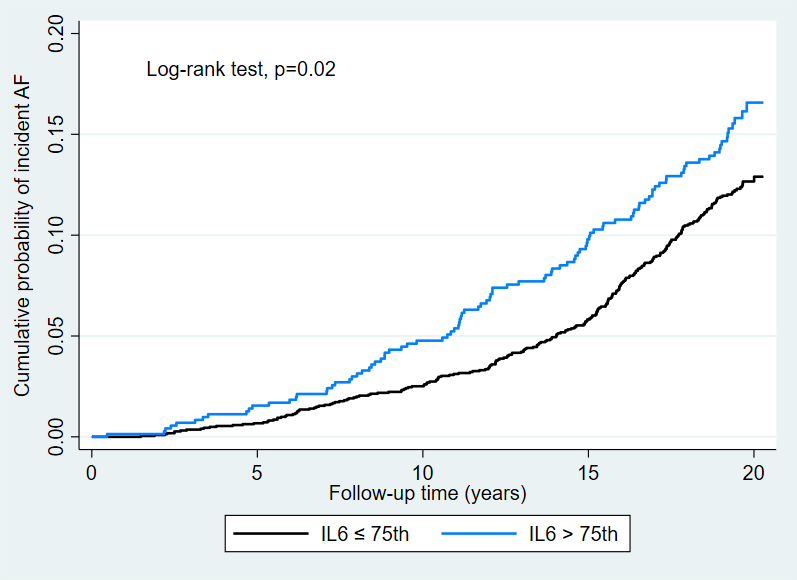


**Additional Figure 2.** Cumulative incidence of atrial fibrillation (AF) in subjects without prevalent AF at baseline. The left panel represents the time to AF diagnosis stratified by IL6 categorized into quartiles and the right panel represents the time to AF stratified by IL6 dichotomized at the 75^th^ percentile.

### **Additional Figure 3.** Cumulative incidence of atrial fibrillation stratified by sIL6R and sgp130


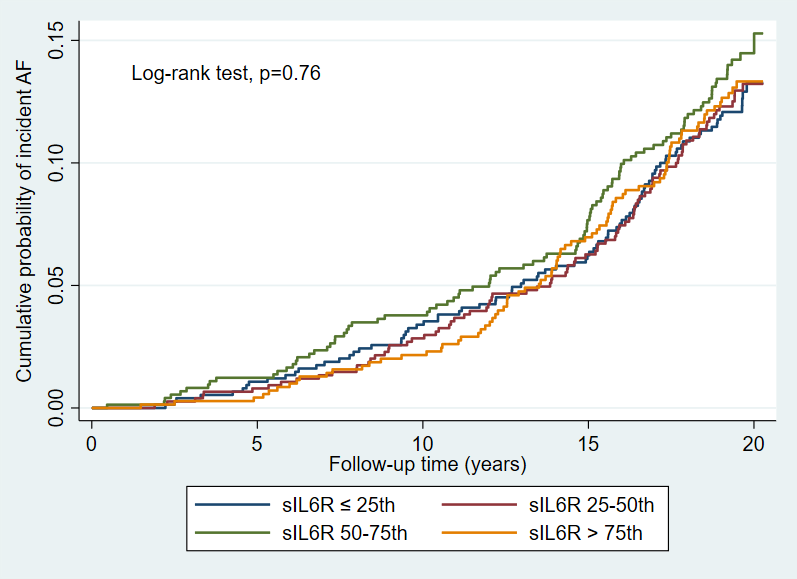

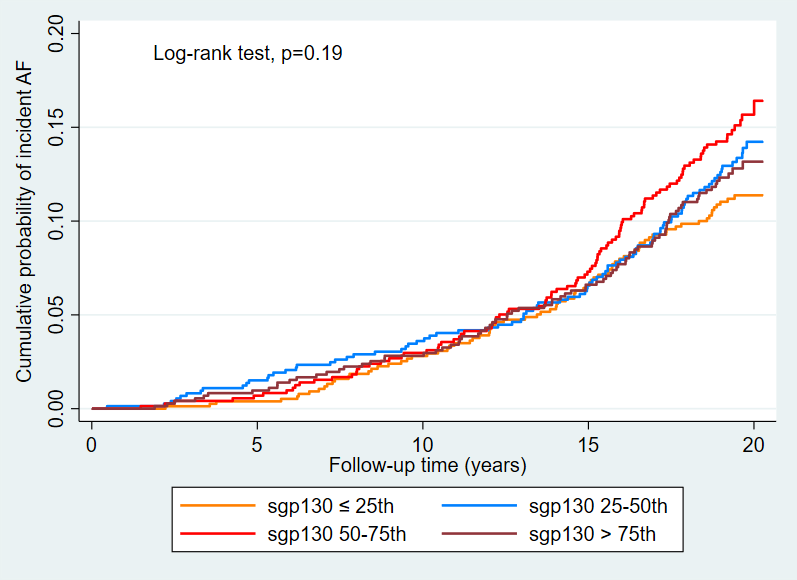


**Additional Figure 3.** Cumulative incidence of atrial fibrillation (AF) in subjects without prevalent AF at baseline. Kaplan Meier failure curves presenting the time to AF diagnosis stratified by the soluble IL6 receptors categorized into quartiles, left panel sIL6R and right panel sgp130.
